# Optical Fiber TFBG Glucose Biosensor via pH-Sensitive Polyelectrolyte Membrane

**DOI:** 10.3390/bios15100642

**Published:** 2025-09-25

**Authors:** Fang Wang, Xinyuan Zhou, Jianzhong Zhang, Shenhang Cheng

**Affiliations:** 1 School of Physics and Optoelectronic Engineering, Taiyuan University of Technology, Taiyuan 030024, China; 2024521678@link.tyut.edu.cn (X.Z.); 2024521667@link.tyut.edu.cn (J.Z.); 2 School of Synthetic Biology, Shanxi University, Taiyuan 030006, China

**Keywords:** glucose biosensor, tilted fiber Bragg grating, pH detection, polyelectrolyte membranes

## Abstract

A novel glucose biosensor is developed based on a tilted fiber Bragg grating (TFBG) functionalized with a pH-responsive polyelectrolyte multilayer membrane, onto which glucose oxidase (GOD) is immobilized. The sensing film is constructed via layer-by-layer self-assembly of poly(ethylenimine) (PEI) and poly(acrylic acid) (PAA), which undergoes reversible swelling and refractive index (RI) changes in response to local pH variations. These changes are transduced into measurable shifts in the resonance wavelengths of TFBG cladding modes. The catalytic action of GOD oxidizes glucose to gluconic acid, thereby modulating the interfacial pH and actuating the polyelectrolyte membrane. With an optimized (PEI/PAA)_4_(PEI/GOD)_1_ structure, the biosensor achieves highly sensitive glucose detection, featuring a wide measurement range (10^−8^ to 10^−2^ M), a low detection limit of 27.7 nM, and a fast response time of ~60 s. It also demonstrates excellent specificity and robust performance in complex biological matrices such as rabbit serum and artificial urine, with recovery rates of 93–102%, highlighting its strong potential for point-of-care testing applications. This platform offers significant advantages in stability, temperature insensitivity, and miniaturization, making it well-suited for clinical glucose monitoring and disease management.

## 1. Introduction

pH represents one of the most critical physicochemical parameters in aqueous systems, serving as a fundamental determinant in numerous natural phenomena, chemical processes, and biochemical reactions [1,2]. Consequently, precise pH monitoring holds substantial significance across diverse fields including agricultural science, environmental monitoring, biomedical engineering, and pharmaceutical development [3,4]. Conventional pH measurement techniques predominantly employ potentiometric methods utilizing glass electrode-based pH meters. However, these conventional systems exhibit several inherent limitations, including prolonged response times, limited measurement accuracy, operational complexity, and incompatibility with remote sensing applications [5]. In contrast, fiber-optic pH sensors have emerged as a promising alternative technology, offering distinct advantages such as enhanced measurement precision, exceptional long-term stability, inherent immunity to electromagnetic interference (EMI), miniaturized form factor, and straightforward fabrication processes [6,7,8,9,10,11]. The development of fiber-optic pH sensors has progressed significantly through various technological approaches. Corres et al. proposed a pH sensing platform in 2007 by immobilizing a polymer nanocoating on long-period fiber grating (LPG) structures, demonstrating effective pH detection within the 4–7 range through theoretical modeling and experimental validation [6]. Yulianti et al. conducted research in 2012 by integrating pH-responsive hydrogels with fiber Bragg grating (FBG) technology [7]. Their system exploited hydrogel swelling dynamics to achieve a characteristic wavelength shift of 49.7 pm across the pH range of 3–9. The 2019 development of an intrinsic optical fiber pH sensor incorporating a polyaniline-polyvinyl alcohol (PANI-PVA) composite coating, which exhibited remarkable sensitivity (2.79 μW/pH unit) across the broad pH range of 2–9 [8]. Lei et al. (2023) demonstrated an advanced tapered fiber-optic sensor configuration based on Mach-Zehnder interferometry (MZI), incorporating chitosan/polyacrylic acid (PAA) polyelectrolytes [9]. This design achieved exceptional pH sensitivity (−27.06 nm/pH) in the physiologically relevant range of 5.0–7.5, while maintaining minimal temperature cross-sensitivity (0.012 nm/°C) within the 25–45 °C range. Beyond interferometric and grating-based architectures, fluorescence-based fiber-optic pH sensors have also gained considerable attention due to their high sensitivity and suitability for in vivo applications. For instance, Zhang et al. developed a miniaturized fluorescence pH sensor with assembly free ball lens on a tapered multimode optical fiber, demonstrating excellent stability and fast response [10]. Additionally, colorimetric and surface plasmon resonance (SPR)-based fiber sensors represent another prominent category. Orouji et al. reported a wide-range pH indicator using gold@silver nanorods, leveraging localized surface plasmon resonance (LSPR) shifts for pH monitoring [4]. In addition, Li et al. proposed a fiber-optic pH sensor based on SPR and polyacrylic acid/chitosan (PAA/CS) self-assembled nanomembrane, achieving high sensitivity and a broad detection range [11]. These advancements highlight the diverse strategies employed in optical pH sensing, each with distinct advantages in terms of sensitivity, dynamic range, and applicability to complex environments. Above all, the fundamental architecture of optical pH sensors typically comprises two essential components: an optical fiber platform serving as the light sensing element, and a functional polymer matrix acting as the pH-responsive sensing medium. This dual-component enables the conversion of chemical information into measurable optical signals through various transduction mechanisms.

The tilted fiber Bragg grating (TFBG) represents a specialized class of fiber Bragg gratings characterized by a deliberate angular offset from the optical fiber axis. This unique configuration establishes an innovative platform for efficient core-to-cladding mode coupling [12,13,14]. TFBGs offer significant advantages including straightforward fabrication, cost-effectiveness, and exceptional sensitivity, making them particularly suitable for high-performance biomedical and chemical sensing applications [15,16,17,18,19,20]. The operational principle of TFBG sensors relies on the coupling of incident light into backward-propagating cladding modes, which subsequently interact with the external medium to induce characteristic spectral modulation [20]. Importantly, TFBGs maintain the fundamental core mode that satisfies Bragg conditions. This dual-mode characteristic enables simultaneous detection of environmental perturbations through the cladding mode while providing a stable reference via core mode monitoring [21]. The core mode spectral shift facilitates compensation for temperature variations and experimental noise, thereby offering TFBG sensors with superior temperature stability and establishing them as ideal candidates for robust fiber optic sensing systems. Recent developments in 2018 and 2024 demonstrated TFBG-based pH sensors incorporating polyaniline (PANI) coatings, achieving pH detection across a broad range (2–12) with a sensitivity of −0.03815 nm/pH through surface plasmon resonance [22,23]. However, the synthesis of PANI typically involves polymerization of formaldehyde and aniline in acidic media, raising concerns due to the inherent toxicity and carcinogenic potential of these precursor materials, which may pose substantial environmental and human health risks. In addition to the direct measurement of pH, numerous sensing platforms incorporate pH converters—functional materials or systems that transduce pH variations into optical, electrical, or mechanical signals. These converters are integral to many biosensing systems, especially those relying on enzymatic reactions that produce pH changes. The development of efficient and stable pH converters is thus crucial for enhancing the performance of pH-mediated biosensors [24,25]. In this work, the polyelectrolyte multilayer serves as both a pH-responsive material and an effective pH-to-optical signal converter, enabling highly sensitive glucose detection.

Polyacrylic acid (PAA) is widely used in many fields due to its non-toxicity, excellent biocompatibility, and low cost, particularly in polyelectrolyte hydrogel pH sensors [26]. Its high hydrophilicity results from carboxyl groups on each unit. Under acidic conditions, most carboxyl anions (-COO^−^) protonate to -COOH, reducing the film’s hydrophilicity [27]. By alternating the deposition of polymer with opposite charges or those having complementary forces, a multilayer film is constructed. Shao et al. developed a TFBG pH sensor incorporating self-assembled poly (diallyldimethylammonium chloride)/PAA multilayer nanocoatings [28]. This configuration demonstrated a near-linear pH response with sensitivity of 117 arbitrary units (a.u.) per pH unit and ±1 a.u. accuracy within the pH range of 4.66–6.02. However, intensity-based demodulation in this sensor shows susceptibility to environmental and light source variations, while the high refractive index (RI) of the coating limits the operational pH range. In the present study, we employ polyethyleneimine (PEI, (CH_2_CH_2_NH)_n_) as an adhesive layer in conjunction with PAA as the functional pH-responsive component. The amine groups in PEI enable formation of hydrogen bonds, ionic interactions, and covalent bonds with hydroxyl groups, while its vinyl groups contribute to strong adhesion properties [29]. Through layer-by-layer (LBL) electrostatic self-assembly (ESA), we fabricated a (PEI/PAA)_n_-pH-sensitive polyelectrolyte membrane on TFBG surfaces. pH-dependent swelling behavior of this polyelectrolyte membrane induces measurable RI changes, which we monitor through wavelength shifts in the TFBG transmission spectrum. Our optimized sensor achieves a linear pH sensitivity of −0.132 nm/pH unit across an extended operational range.

The TFBG-based pH sensor developed in this study demonstrates excellent potential for glucose detection applications. Glucose, a fundamental carbohydrate, serves as both a critical metabolic intermediate and the primary energy source for the human body. Recent years have witnessed growing significance of glucose monitoring across diverse fields, including food industry fermentation processes, as well as in the management of obesity and diabetes mellitus [30,31]. Among various glucose sensing technologies, enzyme-based sensors have gained widespread adoption. In particular, glucose oxidase (GOD) has emerged as the predominant transducing enzyme, catalyzing the conversion of glucose to gluconolactone and hydrogen peroxide (H_2_O_2_) [32,33]. This enzymatic reaction induces an acidic pH shift due to the specific interaction between glucose and GOD, thereby altering the solution pH [34,35]. Consequently, glucose concentration can be quantitatively determined by monitoring the corresponding TFBG wavelength shifts. Through tunable polyelectrolyte membranes, our sensor achieves accurate glucose concentration detection across physiologically relevant concentrations with high specificity, rapid response, and a remarkably low detection limit of 10^−8^ M. The sensor’s miniaturized design, operational simplicity, and resistance to temperature interference collectively address the stringent requirements of modern point-of-care testing (POCT) in medical applications. This innovative platform enables portable, real-time glucose monitoring without geographical constraints, offering substantial practical utility in clinical diagnostics and healthcare management.

## 2. Experimental System and Method

### 2.1. Materials and Reagents

Polyethyleneimine (PEI, branched, Mw ~25,000) and poly(acrylic acid) (PAA, Mw ~100,000) were purchased from Sigma-Aldrich (Shanghai, China). Phosphate-buffered saline (PBS, 10 mM, pH 7.4) and glucose oxidase (GOD, ≥100 U/mg) were obtained from Sheng Gong Co., Ltd. (Shanghai, China). Glucose, fructose and galactose were purchased from Energy Chemical (Shanghai, China). All glucose solutions were prepared in PBS to maintain a stable initial pH and ionic strength. The PBS buffer minimizes the intrinsic pH variation of glucose solutions. The biological samples included rabbit serum and artificial urine. Rabbit serum was obtained from Sheng Gong Co., Ltd. (Shanghai, China). Artificial urine was prepared according to a standard recipe. Different reagents were dissolved in deionized water and the pH was adjusted to 6.0 (9.0 g of NaCl, 3.0 g of Na_2_SO_4_, 0.6 g of NaH_2_PO_4_·2H_2_O, 2.0 g of KCl, 0.9 g of (NH_4_)_2_HPO_4_, 1.7 g of C_4_H_7_N_3_O, 3.0 g of NH_4_Cl, and 25 g of urea were dissolved into 1 L ultrapure water). Deionized water was obtained from ELGA Purelab Option equipment (Paris, France). All chemicals were of analytical grade and supplied by Sinopharm Chemical Re-agent Co., Ltd. (Shanghai, China).

### 2.2. Experimental System

**Figure 1:**
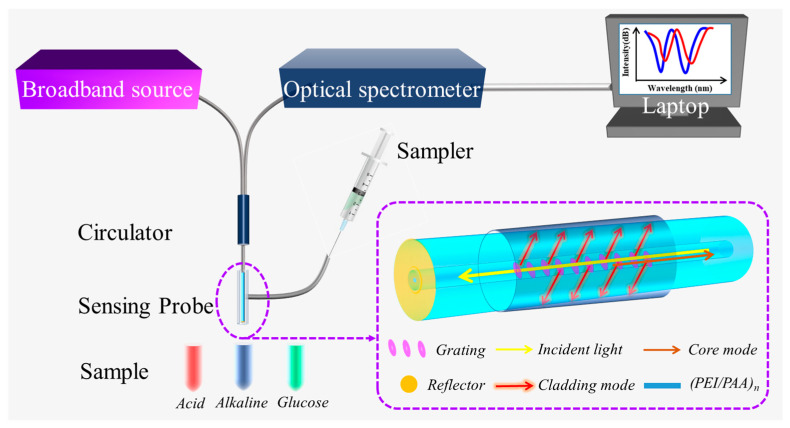
Experimental setup for TFBG-based glucose biosensor.

The experimental configuration, schematically presented in Figure 1, comprises a broadband amplified spontaneous emission source (ASE, Haoyuan Optoelectronic Technology Co., Ltd., Shenzhen, China) an optical circulator, a tilted fiber Bragg grating (TFBG) sensing probe, an optical spectrum analyzer (OSA, Yokogawa AQ6370D, Tokyo, Japan), and a computational workstation. Light emitted from the ASE source is directed through the circulator to the TFBG sensor. This circulator configuration enables reflective-mode operation by separating forward-propagating and backward-propagating optical signals. The resultant spectral output from the sensor is acquired by OSA operating in high-resolution mode (1 pm resolution). To facilitate real-time monitoring, the TCP/IP communication protocol is implemented for remote spectrometer control. Spectral data acquisition and processing are executed via LabVIEW software utilizing NI-VISA algorithms, employing a 10 s spectral integration time. Here, the TFBG was fabricated in a hydrogen-loaded single-mode fiber using the phase mask technique, with the following parameters: grating period = 555 nm, grating length = 10 mm, tilt angle = 4°, Bragg wavelength = 1610 nm [36]. The cladding mode resonances exhibit spectral signatures within the 1565–1605 nm range. The tilt angle of 4° was chosen as it optimizes both the cladding mode density and the coupling strength for refractive index sensing, while also ensuring fabrication feasibility and spectral clarity. As shown in Figure 1, the gold film at the end of the TFBG probe reflects the light from the cladding and the core, with a reflectivity close to 100%. Compared with the transmission sensing system, this structure can effectively reduce the sensor volume and eliminate the influence of stress at the same time. When using the TFBG sensor to detect the glucose concentration, the sensor needs to be placed in a constant-temperature water bath. The temperature of glucose oxidase action is generally 25–60 °C [37]. We control the temperature of the water bath at 38 °C to ensure that the sensor’s detection environment temperature does not change significantly during the detection process. 

### 2.3. Fabrication of Optical Fiber TFBG Glucose Biosensor

**Figure 2:**
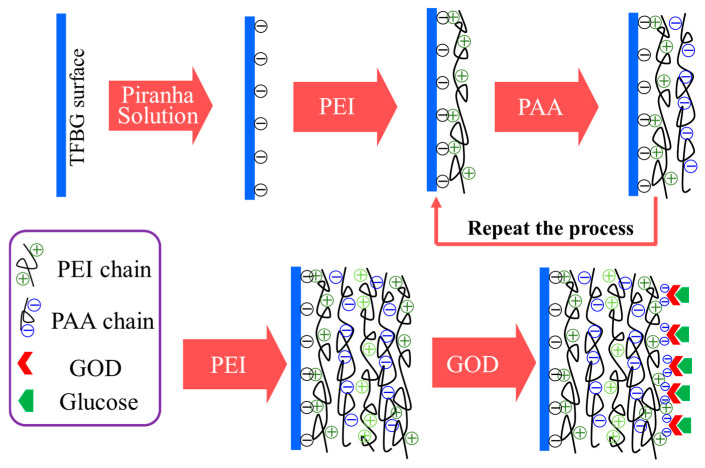
Self-assembly diagram of polyelectrolyte multilayer film.

The optical fiber TFBG glucose biosensor based on pH-sensitive polyelectrolyte membranes were prepared by the chemical cross-linking method as shown in Figure 2. Initially, the TFBG was functionalized by immersion in piranha solution (3:1 *v*/*v* H_2_SO_4_:H_2_O_2_) for 40 min to activate surface hydroxyl groups (-OH), thereby inducing a negative surface charge. Subsequently, the functionalized TFBG was alternately immersed in cationic polyethyleneimine (PEI solution: 2.0 g/L in deionized water, pH adjusted to 11.0 using NaOH.) and anionic poly(acrylic acid) (PAA solution: 2.0 g/L in deionized water, pH adjusted to 3.0 using HCl) solutions for 10 min per layer at ambient temperature. To removed non-adsorbed components and ensure uniform film adhesion, the substrate was rigorously rinsed with deionized (DI) water (1 min) between successive PEI and PAA depositions, followed by nitrogen drying. This process yielded a single bilayer, designated (PEI/PAA)_1_, on the TFBG surface. Iterative deposition achieved the target number of bilayers (PEI/PAA)_n_. Ultimately, the TFBG functionalized with a precisely controlled (PEI/PAA)_4_-PEI multilayer was immobilized in glucose oxidase (GOD) solution to confer biosensing capability. Briefly, the functionalized TFBG with a (PEI/PAA)_4_-PEI multilayer was immersed in a GOD solution (2 mg/mL in PBS, pH 7.4) for 2 h at room temperature to allow physical adsorption and covalent coupling. The sensor was then rinsed with PBS to remove unbound enzyme and dried under a gentle nitrogen stream. Sensors were stored at 4 °C in PBS when not in use. The final biosensor structure is denoted as (PEI/PAA)_4_(PEI/GOD)_1_, indicating four PEI/PAA bilayers followed by one PEI/GOD bilayer. The intermediate structure (PEI/PAA)_4_ describes the intermediate structure before GOD immobilization, which consists of four PEI/PAA bilayers for pH value detection.

GOD could efficiently catalyze the oxidation of β-D-glucose to D-glucono-1,5-lactone, utilizing molecular oxygen as an electron acceptor while producing H_2_O_2_ as a byproduct. The reaction occurs in two primary steps: first, glucose binds to the active site of GOD, where the flavin adenine dinucleotide (FAD) cofactor abstracts two electrons and two protons from the glucose molecule, converting it into gluconolactone and reducing FAD to FADH_2_. Subsequently, the enzyme-bound FADH_2_ transfers the electrons to oxygen (O_2_), regenerating the oxidized FAD cofactor and yielding H_2_O_2_ [37]. The gluconolactone then spontaneously hydrolyzes to gluconic acid in aqueous solutions. The reaction’s dependence on oxygen can be summarized by the following equation:

$$D\text{-}\mathrm{Glucose}+H_{2}O+O_{2}\;\overset{GOD}{\to }D\text{-}\mathrm{GlucoseAcid}+H_{2}O_{2}$$

The efficiency and specificity of GOD arise from its tight binding pocket, which accommodates the pyranose form of glucose while excluding other sugars, ensuring minimal interference in complex matrices [38]. Under the catalysis of GOD, glucose will be oxidized to gluconic acid, which will lead to the change in pH of the solution we measured, and further lead to the change in swelling degree of the fabricated sensing film, which will lead to the deviation of resonance spectrum and the change in resonance coupling strength of the envelope mode.

## 3. Results and Discussion

### 3.1. Spectral Evolution of TFBG with Polyelectrolyte Membranes

**Figure 3:**
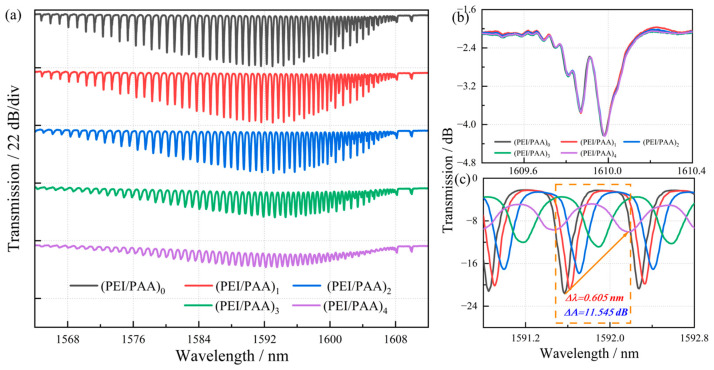
Spectral response of TFBG with (PEI/PAA)_n_ deposition. (**a**) 0~4-layer (PEI/PAA) sensing film spectrum change during film coating. (**b**) Spectral variation of core mode (1610 nm). (**c**) Spectral variation of high-order cladding modes (1591–1593 nm).

Figure 3 illustrates the spectral evolution of TFBG in terms of wavelength shift and intensity modulation as increasing (PEI/PAA) bilayer deposition. TFBGs fundamentally alter light propagation symmetry through phase-matched coupling of core-guided modes to discrete cladding modes at well-defined resonance wavelengths, thereby enabling enhanced light–matter interaction with the ambient medium. The transmission spectrum characteristically exhibits multiple resonance peaks corresponding to excitation of various cladding modes. As demonstrated in Figure 3a, progressive bilayer deposition induces continuous core-to-cladding mode coupling, which significantly attenuates higher-order cladding mode resonances through broadband radiation loss. In contrast, the TFBG’s Bragg resonance (Figure 3b) arises from counter-propagating core mode coupling, with its electromagnetic field tightly confined within the fiber core. Consequently, this Bragg peak remains spectrally invariant to external refractive index perturbations. For quantitative analysis of TFBG’s cladding mode evolution during polymer deposition, we systematically characterized wavelength shifts and intensity variations per bilayer (Figure 3c). The coating process induces two concurrent phenomena: (1) increasing film thickness enhances cladding-mode light absorption, and (2) both real and imaginary components of the effective refractive index experience monotonic growth. The former effect produces characteristic redshift in resonance wavelengths (Δλ = +0.605 nm for (PEI/PAA)_4_), while the latter elevates the extinction coefficient, manifesting as progressive resonance attenuation (ΔA = −11.545 dB) and spectral narrowing. Notably, spectral envelope depression occurs due to radiation mode emergence, a direct consequence of the PEI/PAA multilayer’s elevated refractive index (n = 1.5–1.7) relative to the silica cladding (n ≈ 1.45). Beyond the fourth bilayer, we observe pronounced spectral amplitude reduction and marked wavelength shifting. This nonlinear response profile informed our selection of 4-bilayer coated TFBGs for subsequent glucose sensing experiments, optimizing the trade-off between sensitivity and signal-to-noise ratio. Meantime, this TFBG sensor exhibits inherent temperature self-compensation capability, with a measured temperature cross-sensitivity of <0.01 nm/°C, ensuring reliable performance under physiological temperature variations.

### 3.2. pH-Sensing Performance

**Figure 4:**
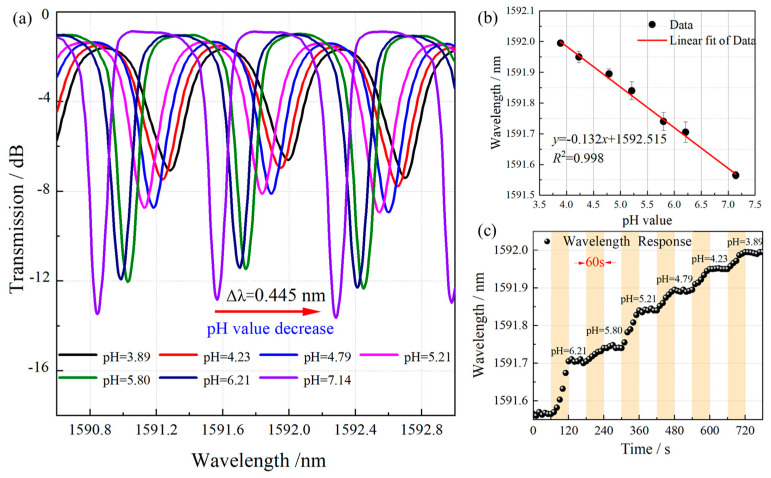
Spectral response of pH-based TFBG biosensor with different pH. (**a**) Local spectral analysis of TFBG with polyelectrolyte membranes in different pH solutions. (**b**) Wavelength changes of TFBG with polyelectrolyte membranes in different pH solutions. Error bars indicate standard deviations of three repeated measurements. (**c**) Continuous monitoring of different pH values and a response time of 60 s.

The pH tests were first conducted by immersing the sensor in standard buffer solutions with known pH values to establish its characteristic sensitivity. The ionic strength was maintained at approximately 0.1 M using PBS. Furthermore, to demonstrate the sensor’s dynamic response in a saline environment, a separate experiment was conducted where the sensor was immersed in 0.9% NaCl solution maintained at 25 °C, with subsequent incremental addition of 0.1 M HCl solution to precisely modulate the solution acidity. Time response was recorded by monitoring the wavelength shift in real-time after each pH change. Figure 4 presents the characteristic spectral responses of the (PEI/PAA)_4_-functionalized TFBG sensor across varying pH conditions. Notably, a consistent redshift in the cladding modes wavelength was observed with decreasing pH values, demonstrating the fundamental pH-responsive behavior that underpins its potential application in glucose detection systems. The pH-dependent response mechanism of (PEI/PAA)_n_ -polyelectrolyte membranes primarily originate from the dissociation equilibrium of carboxylic acid groups and the consequent interplay between electrostatic and hydrophobic interactions. The PAA component contains abundant carboxyl (-COOH) functional groups whose protonation state is pH-dependent. Under acidic conditions, the carboxyl groups remain predominantly protonated (-COOH), rendering the PAA chains electrically neutral. In this state, intramolecular hydrogen bonding and hydrophobic interactions promote chain contraction and the formation of compact hydrophobic domains, significantly reducing polymer solubility. This conformational change enhances the complexation between PEI and PAA, leading to decreased swelling of the multilayer film. The consequent increase in effective refractive index induces both a measurable redshift in the transmission spectrum wavelength and a reduction in spectral intensity (Figure 4a). Furthermore, quantitative analysis revealed a highly linear correlation (R^2^ = 0.998) between wavelength shift and pH value across the physiologically relevant range of 3.89–7.14, as illustrated in Figure 4b. The sensor exhibited a characteristic sensitivity of −0.132 nm/pH unit, demonstrating its potential for precise pH monitoring applications. The response time of the sensor, defined as the time required to reach 90% of the maximum signal change, was approximately 60 s, as illustrated in Figure 4c.

### 3.3. Glucose Detection and Discussion

**Figure 5:**
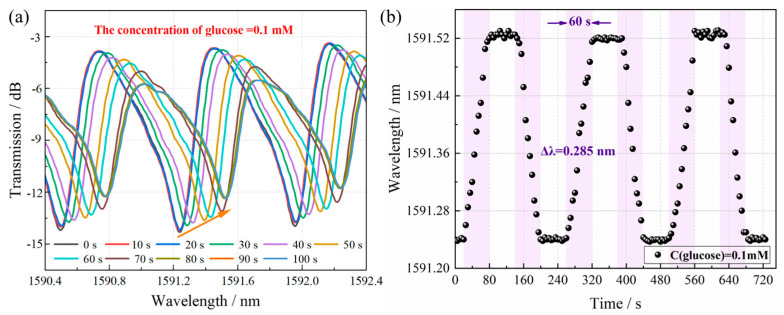
Spectral response of TFBG with (PEI/PAA)_4_(PEI/GOD)_1_ multilayer for glucose detection. (**a**) Spectral analysis of 0.1 mM glucose solution during reaction. (**b**) Response time and Repeatability of TFBG glucose biosensor.

Glucose solutions were prepared by dissolving D-glucose in PBS at concentrations ranging from 10^−8^ to 10^−2^ M. The PBS buffer was used to maintain physiological ionic strength and pH stability during measurements. Then the TFBG biosensor was immersed in the glucose solution held at 38 °C in a temperature-controlled water bath. Spectral data were recorded in real-time using an OSA with LabVIEW-based data processing. The GOD-catalyzed oxidation of glucose generates gluconic acid, thereby inducing a proton concentration gradient that alters the solution pH. This pH variation triggers a conformational change in the polymeric sensing matrix through protonation/deprotonation effects, manifesting as tunable swelling degrees. Such volumetric changes subsequently perturb the photonic resonance conditions, producing both a red-shift in the resonant wavelength and a quantifiable modulation of the envelope mode’s coupling efficiency. Figure 5a presents the spectral analysis of a 4–TFBG glucose sensor functionalized with a (PEI/PAA)_4_(PEI/GOD) sensing layer during exposure to a 0.1 mM glucose solution. The time-resolved spectral data demonstrate a progressive red shift in the TFBG resonance wavelength accompanied by concurrent intensity variations. Notably, the wavelength shift exhibits significantly greater consistency and regularity compared to the intensity changes. These observations confirm that the sensor operates primarily through wavelength modulation, establishing it as a wavelength-modulated glucose detection platform. Response time is operationally defined as the temporal interval between the initial detection of the optical signal by the fiber-optic probe immersed in the glucose solution and the subsequent stabilization of the sensor output. As illustrated in Figure 5b, the enzymatic oxidation of glucose molecules, catalyzed by GOD in the presence of dissolved oxygen, induces a measurable wavelength shift (Δλ = 0.285 nm) in the TFBG cladding mode resonance peak. And the response time of the sensor is about 60 s. In addition, the sensor exhibits good repeatability with a standard deviation of ±0.01 nm and reversible behavior enabling continuous monitoring of dynamic glucose concentration changes. After each measurement, the sensor can be refreshed by rinsing with PBS buffer, which restores the baseline signal within 1 min. This reversibility is attributed to the pH-responsive swelling/deswelling dynamics of the PEI/PAA polyelectrolyte multilayer, which returns to its initial state upon removal of gluconic acid.

**Figure 6:**
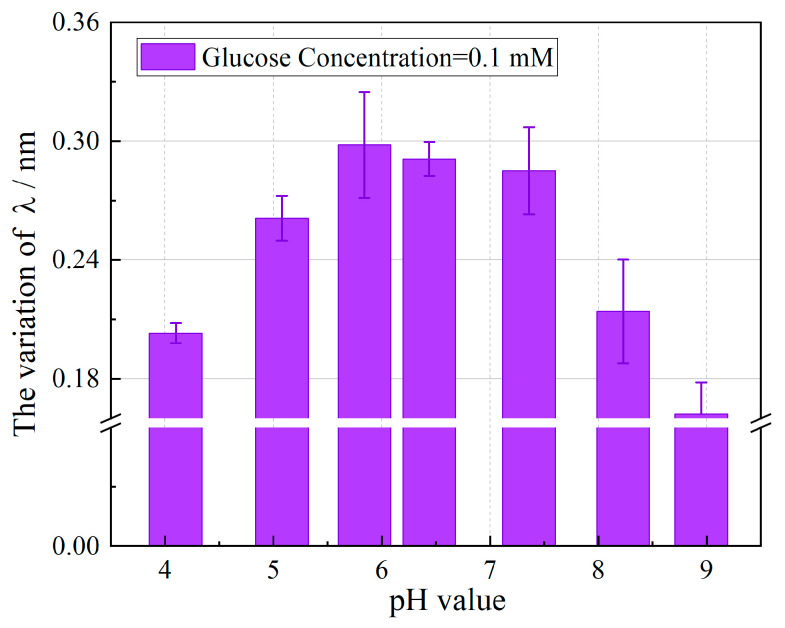
Wavelength response of TFBG-based glucose biosensor at different pH values.

It is widely recognized that pH exerts a profound influence on enzymatic activity, raising a fundamental question regarding how the intrinsic pH of real samples affects glucose detection. To address this, the pH-dependent response of the sensor was systematically evaluated. A series of PBS solutions containing a fixed glucose concentration of 0.1 mM were prepared, with pH adjusted from 4.0 to 9.0 using 0.1 M HCl or NaOH. The sensor’s response, quantified as the wavelength shift (Δλ), was recorded at each pH level. As depicted in Figure 6, the sensor demonstrated consistent and measurable responsiveness to glucose across the entire pH range tested. Under acidic conditions, Δλ increased progressively with pH, attaining a maximum at pH 5.8. Beyond this optimum, the response gradually decreased with the further elevation in pH, a behavior attributed to the reduced protonation of carboxyl groups in PAA under alkaline conditions. The optimal pH for the enzymatic reaction between GOD and glucose is typically within the range of 5.5–6.5. Highly acidic conditions may lead to irreversible enzyme denaturation and consequent loss of catalytic activity, while alkaline environments can disrupt enzyme structure and promote side reactions such as isomerization, thereby reducing reaction efficiency. Despite these limitations, the sensor maintained detectable responsiveness even at higher pH values, highlighting its robustness across varying pH conditions. These results indicate that although the absolute sensor response is influenced by ambient pH, the enzymatic production of gluconic acid remains sufficient to induce a measurable local pH change at the sensor interface. This affirms the potential applicability of the sensor in real-world samples with inherent pH variability. In this study, a pH of 7.4 was selected for the PBS buffer to simulate physiological conditions.

**Figure 7:**
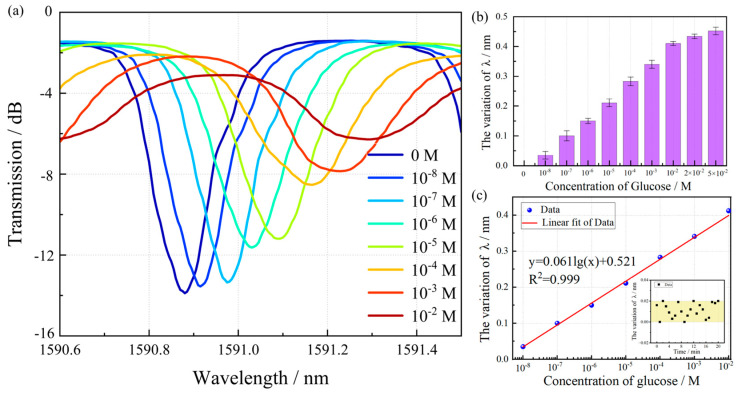
Spectral response for different glucose concentrations. (**a**) Transmission spectra of the biosensor with the D-glucose concentration in the PBS solution from 10^−8^ M to 10^−2^ M. (**b**) Resonant peak shifts of the biofunctionalized TFBG sensor with the D-glucose concentration range of 10^−8^−5 × 10^−2^ M (Error bars indicate standard deviations of measurements by three sensors). (**c**) The LOD and blank standard deviation of glucose sensor.

The physiological blood glucose concentration in humans is strictly regulated and typically does not exceed 10^−2^ M. To investigate the detection of saccharide molecules, we prepared a series of glucose solutions with concentrations ranging from 10^−8^ to 10^−2^ M. Initially, the transmission spectrum of the biosensor immersed in pure PBS solution (0 M glucose) was acquired as a baseline reference. Upon enzymatic reaction with glucose oxidase (GOD), a characteristic red shift in the spectral wavelength was observed, with the magnitude of this shift exhibiting a positive correlation with increasing glucose concentration. This phenomenon can be attributed to the following mechanism: Elevated glucose concentrations lead to enhanced production of gluconic acid during the enzymatic reaction, resulting in a progressive decrease in solution pH. Under acidic conditions, the carboxyl groups (-COO^−^) in the PAA layer undergo protonation to form -COOH. This transformation reduces the overall hydrophilicity of the multilayer film, thereby strengthening the polyelectrolyte complex between PEI and PAA. Consequently, the degree of film swelling decreases, leading to an increase in the effective refractive index and the observed red shift in the resonance wavelength. As demonstrated in Figure 7a,b, a significant resonance peak shift of 0.412 nm was recorded when the glucose concentration increased from 0 M to 1.0 × 10^−2^ M. The sensor’s response mechanism is specifically dependent on pH-induced swelling of the PAA layer in the inner film, which subsequently alters the surface refractive index of the TFBG. Due to the thickness of the sensing film, the sensor has a threshold phenomenon for pH detection. It may cause saturation in a large range of glucose concentrations (10–50 mM). In the experiment, we fabricated three sensors under identical conditions and tested each with glucose solutions ranging from 10^−8^ M to 5 × 10^−2^ M. Sensor-to-sensor reproducibility was within 2% RSD (in Figure 7b), attributable to minor variations in film thickness and enzyme loading during fabrication. The limit of detection (LOD), defined as the minimum analyte concentration that can be statistically distinguished from the blank value with 95% confidence (typically corresponding to a signal-to-noise ratio of 3), serves as a critical parameter for evaluating method sensitivity. This metric is influenced by multiple factors including instrumental precision, sample matrix effects, and experimental variability. As shown in Figure 7c, a linear correlation was established between the wavelength shift and the logarithmic glucose concentration (10^−8^ M–10^−2^ M). Based on uncertainties of 0.02 nm estimated under PBS buffer, the LOD was calculated as 3 times the standard deviation of the blank signal divided by the slope of a standard curve, yielding a value of 27.7 nM.

**Figure 8:**
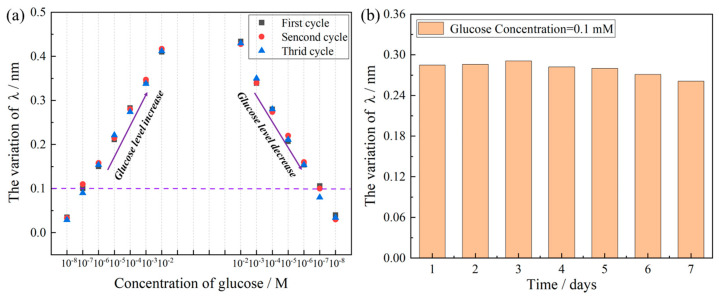
Recovery and stability of TFBG-based glucose biosensor. (**a**) Three cycles of glucose detection between low (10^−8^ M) and high (10^−2^ M) glucose concentrations. (**b**) The long-term stability of TFBG glucose biosensor.

Excellent reversibility and stability are critical for the practical application of biosensors in continuous monitoring. As shown in Figure 8a, the TFBG glucose biosensor exhibits highly reversible performance over three consecutive cycles of alternating exposure to low (10^−8^ M) and high (10^−2^ M) glucose concentrations. The sensor demonstrates consistent and reproducible wavelength shifts with minimal signal attenuation or hysteresis. This reversibility originates from the robust pH-responsive behavior of the PEI/PAA polyelectrolyte multilayer, which undergoes reversible swelling and deswelling in response to local pH changes induced by the enzymatic reaction. The rapid and complete signal recovery upon rinsing with PBS (within ~1 min) further confirms the stability of the immobilized glucose oxidase and the structural integrity of the self-assembled film. Such cyclic stability is essential for continuous monitoring applications requiring repeated analyte exposure without performance degradation. Figure 8b illustrates the long-term stability of the biosensor over 7 days of storage in PBS at 4 °C, with daily testing using a 0.1 mM glucose solution. The sensor retains over 92% of its initial response, demonstrating outstanding operational and storage stability. This prolonged functionality is attributed to the effective encapsulation of GOD within the PEI/PAA matrix, which provides a biocompatible microenvironment that preserves enzymatic activity and prevents denaturation and leaching. Covalent and electrostatic interactions between the enzyme and the polyelectrolyte layers further enhance the mechanical durability of the sensing film. The minimal drift in sensor response over time underscores its reliability for long-term real-world applications. The combination of excellent reversibility and long-term stability makes the TFBG-based biosensor a highly promising platform for continuous glucose monitoring in complex biological fluids. Additionally, the wavelength-based demodulation strategy improves robustness against intensity fluctuations caused by ambient light or source power variations.

**Figure 9:**
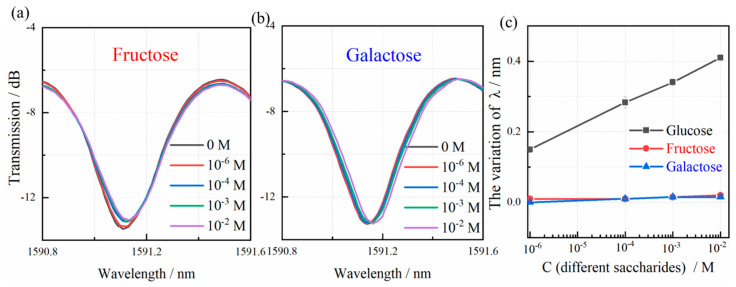
Transmission spectra of the biosensor when the different concentrations of fructose (**a**) and galactose (**b**) were added into the PBS. (**c**) Resonant peak shifts of the biosensor with glucose, fructose and galactose. Error bars indicate standard deviations of three repeated measurements.

Table 1 summarizes glucose detection methodologies as reported in recent studies [39,40,41,42,43]. In clinical diagnostics, fasting blood glucose levels in healthy individuals typically fall within the physiological range of 3.90–6.00 mM (with a diagnostic threshold of ≤10 mM), whereas diabetic patients consistently demonstrate concentrations surpassing 7.0 mM [44]. Quantitative analyses establish the reference range for urinary glucose at <2.8 mM. Significantly, the developed biosensor exhibits exceptional sensitivity, rapid response kinetics, and an extensive linear detection range that fully encompasses these clinically significant glucose concentrations in both blood and urine matrices. Interference resistance represents a critical performance parameter for biosensors. Systematic evaluation of potential cross-reactivity was performed by analyzing the transmission spectra of fructose and galactose solutions across a concentration gradient. Fructose and galactose solutions were prepared in PBS (10 mM, pH 7.4) at concentrations from 10^−6^ to 10^−2^ M. As illustrated in Figure 9a–c, the resonance wavelength shifts induced by fructose and galactose were negligible (<0.02 nm) compared to the PBS control, while glucose solutions produced significant resonance peak displacements. To assess the sensor’s performance in biologically relevant environments, validation experiments were conducted using rabbit serum and synthetic urine matrices. The rabbit serum was diluted in a PBS solution at 5 times, and artificial urine was prepared according to a standard recipe [45]. Prior to the experiments, both rabbit serum and artificial urine samples were brought to room temperature and gently mixed to ensure homogeneity. Known amounts of glucose standard solution were spiked into the rabbit serum and artificial urine samples to achieve final concentrations of 2.00 mM, 4.00 mM, and 6.00 mM. Each spiked sample was thoroughly vortexed for 30 s to ensure uniform distribution of glucose. The biosensor was immersed in the spiked samples, and the resonance wavelength shift was recorded after stabilization. Recovery tests, defined as the ratio of measured to spiked glucose concentrations in real samples, yielded recovery rates ranging from 93% to 102% across various concentrations (Table 2). These findings collectively validate the sensor’s superior specificity, measurement precision, and reliability for glucose quantification in complex biological fluids.

When analyzing actual biological samples (e.g., serum, plasma, urine), the complex and variable composition of these matrices may lead to non-specific physical deposition on the sensor surface. However, such deposits primarily accumulate on the outer sensing film without interacting with the glucose oxidase (GOD) immobilized on the sensor surface. Importantly, these surface deposits demonstrate negligible effects on both the swelling behavior of the underlying PEI/PAA layer and the evanescent wave propagation along the optical fiber’s inner PEI/PAA interface. Furthermore, our detection protocol incorporates blank signal correction using a reference sensor, enabling precise compensation for background interference. The final analytical signal is obtained by subtracting this background noise from the total spectral response, thereby isolating the specific signal arising from the GOD-glucose enzymatic reaction. These methodological considerations suggest that the proposed sensor may offer superior accuracy and reliability for practical glucose monitoring applications. The combination of appropriate linear range, excellent recovery characteristics, and effective interference compensation positions this technology as a promising candidate for clinical glucose detection.

## 4. Conclusions

In this study, we present a novel glucose biosensor utilizing a tilted fiber Bragg grating (TFBG) platform functionalized with a polyelectrolyte multilayer film fabricated through layer-by-layer self-assembly. The sensing mechanism relies on pH-induced modulation of the complexation strength between PEI and PAA within the multilayer structure, which subsequently alters the effective refractive index of the sensing film. This pH-responsive polymeric film demonstrates excellent compatibility with the TFBG platform, enabling highly sensitive pH measurements in solutions (−0.132 nm/pH unit, 3.89–7.14). The glucose biosensor was fabricated by sequentially depositing PEI/PAA multilayers on the TFBG surface, followed by immobilization of glucose oxidase (GOD) as the recognition element. The enzymatic reaction between GOD and glucose generates local pH changes at the sensor interface, which are transduced into optical signals through the TFBG platform. Systematic optimization yielded a linear response (R^2^ > 0.99) between the wavelength shift and the logarithmic glucose concentration (range of 10^−8^–10^−2^ M) and the LOD was 27.7 nM, encompassing typical glucose levels in clinical settings. The developed biosensor combines several advantageous features: high sensitivity to physiologically relevant glucose concentrations, inherent temperature self-compensation capability due to the TFBG architecture, and compact form factor suitable for point-of-care testing (POCT) applications. These attributes make the TFBG-based glucose biosensor a promising candidate for clinical diagnostics and personalized healthcare monitoring, particularly in scenarios requiring rapid, reliable, and minimally invasive glucose measurement.

## Figures and Tables

**Table 1:** Comparison of the LOD and detection range of the biosensor in this article with other reported sensors.

Methods	LOD	Detection Range	Response	Ref.
LPG-(PEI/PAA)_4_(PEI/GOD)_1_	1 nM	1 nM–10 µM	70 s	[39]
Fiber Optic -PNIPAAm-GOD	11.4 mg/dL	50–700 mg/dL	200 s	[40]
Fiber-Fluorescent-CQDs-GOD/CA	25.79 nM	10 nM–200 μM	~10 s	[41]
EX-TFG-AuNPs-GOD	2.5 nM	1 nM–5 mM	55 s	[42]
TFBG-SPR- Electrospinning-GOD	100 mg/dL	100–500 mg/dL	30 s	[43]
TFBG -(PEI/PAA)_4_(PEI/GOD)_1_	27.7 nM	10 nM–10 mM	60 s	This work

**Table 2:** Determination of glucose in real samples using the TFBG glucose biosensor with pH-sensitive polyelectrolyte membrane (The Accuracy is calculated as Accuracy = 100% − ∣100% − Recovery∣, which provides a clear measure of how close the measured value is to the true value).

Sample	Glucose Added (mM)	Glucose Found (mM)	Recovery (%)	Accuracy (%)
Rabbit serum	2.00	2.03	101.50	98.50
	4.00	3.89	97.25	97.25
	6.00	6.11	101.83	98.17
Artificial urine	2.00	1.87	93.50	93.50
	4.00	4.13	103.25	96.75
	6.00	5.99	99.83	99.83

## Data Availability

Data is contained within the article.
